# HIV Prevention in High-Risk Women in South Africa: Condom Use and the Need for Change

**DOI:** 10.1371/journal.pone.0030669

**Published:** 2012-02-17

**Authors:** Francois van Loggerenberg, Alexis A. Dieter, Magdalena E. Sobieszczyk, Lise Werner, Anneke Grobler, Koleka Mlisana, for the CAPRISA 002 Acute Infection Study Team

**Affiliations:** 1 Centre for AIDS Programme of Research in South African (CAPRISA), University of KwaZulu-Natal, Durban, South Africa; 2 Columbia University College of Physicians and Surgeons, Columbia University Medical Center, New York, New York, United States of America; 3 Department of Medicine, Division of Infectious Diseases, Columbia University College of Physicians and Surgeons Columbia University Medical Center, New York, New York, United States of America; University of California, Berkeley, United States of America

## Abstract

**Introduction:**

Young women are at disproportionate risk of HIV infection in South Africa. Understanding risk behaviors and factors associated with ability to negotiate safe sex and condom use is likely to be key in curbing the spread of HIV. Traditionally prevention efforts have focused on creating behavioral changes by increasing knowledge about HIV/AIDS.

**Methods:**

This was a cross-sectional analysis from a prospective observational cohort study of 245 women at a high-risk of HIV infection in KwaZulu-Natal, South Africa.

**Results:**

Participants demonstrated a high level of HIV/AIDS knowledge. Overall, 60.3% of participants reported condom use. Reported condom use at last sexual encounter varied slightly by partner type (57.0% with steady versus 64.4% with casual partners), and self-perceived ability to choose to use a condom was significantly lower with steady partners compared to casual partners (p<0.01). In multivariate analysis, women who had high school education were more likely to use condoms at their last sex encounter compared to those with only primary school education (RR of 1.36 (95% Confidence Interval (CI) 1.06–1.75) and 1.46 (95% CI 1.13–1.88) for grades 8–10 and 11–12, respectively). Those who used condoms as a contraceptive method were twice as likely to use condoms compared to women who did not report using them as a contraceptive method. Greater perceived ability to choose to use condoms was associated with higher self-reported condom use at last encounter, irrespective of partner type (RR = 2.65 (95% CI 2.15–32.5).

**Discussion:**

Self-perceived ability to use condoms, level of formal education and condom use as a contraceptive were all significantly associated with self-reported condom use at last sexual encounter. These findings suggest that that gender inequality and access to formal education, as opposed to lack of HIV/AIDS knowledge, prevent safer sexual practices in South Africa.

## Introduction

In the South African province of KwaZulu-Natal, an estimated 25.8% of all persons aged 15 to 49 years are HIV-positive. This prevalence rate is one of the highest in South Africa, a country that carries the highest number of HIV-infected persons in the world [1], [2]. HIV is predominantly spread via heterosexual sex, with young women being the country's most vulnerable population [1], [3].

Traditionally prevention efforts have focused on creating behavioral changes (i.e. safer sexual practices) within the general population by increasing knowledge about HIV/AIDS. Studies in the United States and other developed countries have demonstrated successful reduction in HIV infection rates with educational campaigns, while South Africa has continued to experience a rise in the prevalence of HIV despite increased public knowledge and awareness of HIV/AIDS [1], [4], [5]. Recent reductions in HIV incidence in South Africa have instead been attributed to increased access to HIV treatment [6]. Additionally, several studies have found that improved access to information about HIV risk is not translating into an effective behavioral change in women at high-risk of infection [1], [7], [8], [9], [10], [11], [12], [13], [14]. Researchers have investigated the possible causes of this disconnect and one theory is that behavioral changed is impeded by the ‘social context’ of this community [15].

Reported level of condom use has been used as way of identifying and understanding barriers to behavioral change. Reported rates of condom use among sexually active populations in South Africa range from 32.8% to 78.4% [1], [16], [17], [18], [19], [20], [21]. In high-risk women, such as commercial sex workers, the rate of condom use is low [13], [14], [15]. The negative stigma associated with condoms, lack of female power in sexual relationships, threat of physical violence if condom use is requested, and financial incentives to forego condom use are all potential barriers to their use [3], [7], [11], [12], [13], [14], [15], [20], [22], [23], [24], [25], [26], [27], [28].

This investigation, as part of a larger behavioral study, focused on women at high-risk of contracting HIV/AIDS. It aimed to examine the impact of HIV/AIDS knowledge, as well as related sexual practices on self-reported condom use, and the influence these factors have on HIV acquisition in a high-risk population.

## Methods

This study was a cross-sectional analysis of baseline behavioral data gathered from women enrolled into a prospective observational cohort study performed at the Centre for the AIDS Programme of Research in South Africa (CAPRISA), in Durban, KwaZulu-Natal Province, South Africa. This investigation, known as the CAPRISA 002 Acute Infection Study, began in 2004 to research the natural history of acute HIV infection and associated risk behaviors in a cohort of high-risk women, 78.8% of who were self-identified sex workers.

CAPRISA 002 study enrolled women > = 18 years of age who self-reported sex with more than three different partners in the previous three months, and who were HIV antibody negative on screening with rapid tests (first with the Determine test (Abbott Laboratories, Tokyo, Japan) and, if positive, confirmed with a Capillus test (Trinity Biotech, Jamestown, NY, USA)). Discordant rapid HIV tests were resolved with a confirmatory HIV enzyme immunoassay (BEP 2000; Dade Behring, Marburg, Germany). More detailed information regarding CAPRISA 002 study methodology, including recruitment methods and eligibility criteria, is described elsewhere [29].

Prior to study initiation, the protocol and informed consent forms were reviewed and approved by the research ethics committees of the University of KwaZulu-Natal (E013/04), the University of Cape Town (025/2004), and the University of the Witwatersrand (MM040202). Consent forms and information to patients were available in English and were translated into isiZulu, the local language. Written informed consent was obtained at each phase of the study, including collecting the behavioral risk data and to store the study specimens.

Participants completed a structured, in-person interview in either English or isiZulu (per participant preference) to identify their HIV risk behaviors. Upon enrollment, demographic, locator information and laboratory samples were obtained from each participant who received HIV pre- and post-test counseling, HIV and sexually transmitted infection (STI) risk reduction counseling, a behavioral risk assessment as well as a routine clinical evaluation. During the administration of the HIV/AIDS knowledge questions, participants were not prompted, but were asked to state their responses to the questionnaire administrator who ticked off the appropriate category according to the participant's response. Responses were then labeled with a participant identification number and entered into a data collection system. Each participant was paid for her participation in the study as a means of reimbursement for their time and travel expenses.

Descriptive and statistical analysis was performed using SAS software version 9.2 (SAS Institute Inc., Cary). Unadjusted and adjusted risk ratios, measuring the associations between the factors of interest, were calculated using generalized estimating equation regression models with the log link function. Variables in the unadjusted model with a p-value less than 0.20, or variables deemed as important to adjust for, were fitted to the multivariate model. The McNemar test was used to assess differences in perceived choice of condom use between participants' casual and steady partners.

## Results

A total of 245 participants were enrolled in the study, the basic characteristics of whom are presented in Table 1. This was a cohort of high-risk women; 85.4% reported having 2–5 casual partners in the 3 months prior to enrollment and 78.8% self-identified as sex workers. Overall, 58.8% of women reported condom use at their last sexual encounter.

**Table 1:** Participant demographic characteristics.

**Mean age in years ± SD**	34.2±10.5
**High school education % (n)**	
- Primary	24.5% (60)
- Secondary to 10^th^ grade	34.3% (84)
- Grades 11/12	41.2% (101)
**Partner status % (n)**	
- No partner	7.4% (18)
- One partner	35.7% (87)
- Many Partners	57.0% (139)
**Race % (n)**	
- Black	98.4% (241)
- Indian	0.4% (1)
- White	1.2% (3)

All participants reported that they had heard of HIV/AIDS at the time of study enrollment and, overall, participants demonstrated a good level of knowledge of HIV disease. These data are presented in Table 2.

**Table 2:** HIV/AIDS awareness and knowledge.

**Have you ever heard of HIV/AIDS? % (n)**	
- Yes	100% (n = 245)
- No	0%
**How is HIV/AIDS spread? % (n)**	
- Vaginal sex	99.6% (n = 244)
- Contact with blood	94.7% (n = 232)
- Anal sex	56.7% (n = 139)
- Oral sex	51.8% (n = 127)
- From mother to child	48.2% (n = 118)
- Breastfeeding	10.6% (n = 26)
- Touching someone	4.9% (n = 12)
- Mosquito bites	4.5% (n = 11)
- Sharing same cup	3.7% (n = 9)
- Using public toilets	2.9% (n = 7)
- Other	62.4% (n = 153)
**How can you prevent the spread of HIV/AIDS? % (n)**	
- Using condoms	99.6% (n = 244)
- Avoid sharing razors	75.1% (n = 184)
- Injections with clean needles	53.5% (n = 131)
- Abstaining from sex	39.2% (n = 96)
- Staying faithful	29.8% (n = 73)
- Having good diet	2.0% (n = 5)
- Other	35.5% (n = 87)
**If you have an STI, do you think you are more likely to get HIV/AIDS? % (n)**	
- Yes	71.8% (n = 176)
- No	1.6% (n = 4)
- Unsure	26.5% (n = 65)
**Do you think that HIV/AIDS can be cured? % (n)**	
- Yes	4.1% (n = 10)
- No	91.4% (n = 224)
- Unsure	4.5% (n = 11)
**Do you think that HIV/AIDS can be treated? % (n)**	
- Yes	95.1% (n = 233)
- No	2.0% (n = 5)
- Unsure	2.9% (n = 7)
**Risk of HIV acquisition by route** **Anal sex compared to penovaginal sex? % (n)**	
- Less risk	30.3% (n = 74)
- Same risk	27.9% (n = 68)
- More risk	34.0% (n = 83)
- Don't know	7.8% (n = 19)
**Oral sex compared to penovaginal sex? % (n)**	
- Less risk	23.9% (n = 58)
- Same risk	33.3% (n = 81)
- More risk	38.3% (n = 93)
- Don't know	4.5% (n = 11)

Participants were knowledgeable about how HIV/AIDS is spread; almost all participants listed vaginal sex as a risk factor, but considerably smaller proportion listed anal or oral sex. When asked about the risk of contracting HIV via anal and oral versus penovaginal sex, slightly more than a third of participants answered that anal and oral sex carried more risk compared to vaginal sex.

Similarly, there was high level of awareness how to prevent acquisition or transmission of HIV. Almost all of the participants listed condoms as an effective method, while only 39% and 30% indicated abstinence and staying faithful to one partner as an effective HIV prevention method. Even though treatment was not widely available at the time of the interviews, the majority of participants knew that HIV could not be cured, while a similar proportion agreed that it could be treated.

Sexual behavior data is presented in Table 3, and indicate that participants were much more likely to have had sex with a steady partner than a casual partner (defined as one seen only occasionally, or even once, that was not a sex work client) at last encounter (75.9% steady versus 24.1% casual). In the three months prior to enrollment, over 85% of participants reported two to five casual partners. With respect to total lifetime partners, 54.1% reported more than five steady partners and 76.1% reported more than five casual partners. Participants reported that they engaged in penovaginal sex an average of 10 times a month, with low levels of oral and anal sex. Approximately 80% of participants reported that they used contraceptives, with 60.3% listing condoms as a method of contraception. Importantly, none of these baseline characteristics or sexual practices were found to be statistically associated with subsequent HIV acquisition.

**Table 3:** Sexual behavior.

Mean age at sexual debut ± SD	17.0±2.4
Mean days since last encounter ± SD	4.6±4.6
**Last sex act partner type % (n)**	
- With casual partner	24.1% (59)
- With steady partner	75.9% (186)
**Number of casual partners in the last 3 months % (n)**	
- 0–1	5.0% (12)
- 2–5	85.4% (205)
- >5	9.6% (23)
**Number of lifetime casual partners % (n)**	
- 0–1	0.0% (0)
- 2–5	23.9% (57)
- >5	76.1% (181)
**Number of steady partners (last 3 months) % (n)**	
- 0–1	68.4% (165)
- 2–5	29.1% (70)
- >5	2.5% (6)
**Number of lifetime steady partners % (n)**	
- 0–1	4.1% (10)
- 2–5	41.7% (101)
- >5	54.1% (131)
Mean number of penovaginal sex acts per month ± SD	10.2±6.7
Mean number of oral sex acts per month % (n)	
- 0–1	79.9% (191)
- 1–4	14.2% (34)
- >4	5.9% (14)
Anal sex acts per month	
- Never	66.8% (159)
- Once or less	11.3% (27)
- ≥2	21.9% (52)
Douche after/between sex % (n)	
- Yes	9.4% (23)
- No	90.6% (221)
Proportion who ever had sex while drunk % (n)	26.9% (66)
Proportion who use any contraceptives % (n)	79.9% (195)
Proportion who use condoms % (n)	60.3% (147)
Contraceptives used: % (n)	
- Condom	32.0% (78)
- Dual (condom+other)	28.3% (69)
- Non-condom	19.3% (47)
- None	20.5% (50)
Proportion using injectable contraceptives % (n)	27.9% (68)

While frequency of contraceptive use was not assessed, condom use at the most recent sexual encounter varied depending on partner type (see Table 4). For example, 57.0% of participants reported using a condom with a steady partner, compared to 64.4% with a causal partner (p = 0.36). Perceived ability to negotiate condom use with casual partners was significantly higher compared to steady partners, with only 20.8% of participants saying they felt they could always use condoms with their steady partner versus 53.9% with casual partners (p<0.01).

**Table 4:** Condom use.

Condom used at last sexual encounter % (n)	58. 8% (144)	
- Steady partner at last sexual encounter: condom use % (n)	57.0% (106/186)	Fisher's exact
- Casual partner at last sexual encounter: condom use % (n)	64.4% (38/59)	p = 0.36
**Perceived ability to choose condom use with steady partner % (n)**		
- Never	35.0% (84)	
- Sometimes	44.2% (106)	
- Every time	20.8% (50)	
**Perceived ability to choose condom use with casual partner % (n)**		
- Never	13.9% (34)	
- Sometimes	32.2% (79)	McNemar
- Every time	53.9% (132)	p<0.01

Several factors were associated with condom use at last sexual encounter. For example, self-perceived ability to choose to use condoms was associated with self-reported use both in univariate and multivariate analyses (see Table 5). In the multivariate model, women who believed they had a high degree of control over condom use (able to choose always or over half the time whether or not to use condoms) were more than twice as likely to have used a condom than those who reported never having the ability to choose or who felt they could only insist on using a condom less than half the time (RR 2.21, 95% CI 1.79–2.72).

**Table 5:** Perceived ability to choose to use a condom and predictors of condom use at last sexual encounter.

Variable	Univariate analysis			Multivariate analysis	
	% with condom use at last sex act (n/N)	Risk Ratio (95% CI)	p-value	Risk Ratio (95% CI)	p-value
Ability to insist on condom use (number of times)					
- Less than half/Never	36.9% (58/157)	1.00 (reference)	-	1.00 (reference)	-
- More than half/Always	97.7% (86/88)	2.65 (2.15–3.25)	**<0.01**	2.21 (1.79–2.72)	**<0.01**
Age (by 5 year increase)		0.92 (0.88–0.97)	**<0.01**	0.99 (0.94–1.04)	0.69
Age at sexual debut (by 5 year increase)		0.98 (0.78–1.24)	0.88		
Schooling					
- No schooling	28.6% (2/7)	1.00 (reference)	-		
- Any schooling	59.7% (142/238)	2.09 (0.64–6.77)	0.22		
Highest level of school					
- Primary	43.3% (26/60)	1.00 (reference)	-	1.00 (reference)	-
- Grade 8–10	63.1% (53/84)	1.46 (1.04–2.03)	**0.03**	1.36 (1.06–1.75)	**0.02**
- Grade 11–12	64.4% (65/101)	1.49 (1.07–2.05)	**0.02**	1.46 (1.13–1.88)	**<0.01**
Marital status					
- One partner	59.8% (52/87)	1.00 (reference)	-	1.00 (reference)	-
- Many partners	56.1% (78/139)	0.94 (0.75–1.18)	0.59	0.92 (0.76–1.10)	0.36
- No partner	72.2% (13/18)	1.21 (0.86–1.69)	0.27	1.39 (1.01–1.90)	**0.04**
Last sexual partner					
- Steady	57.0% (106/186)	1.00 (reference)	-	1.00 (reference)	-
- Casual	64.4% (38/59)	1.13 (0.90–1.42)	0.29	1.05 (0.87–1.26)	0.61
Sex worker					
- No	69.2% (36/52)	1.00 (reference)	-	1.00 (reference)	-
- Yes	56.0% (108/193)	0.81 (0.65–1.01)	0.06	0.96 (0.78–1.18)	0.69
Ever had anal sex?					
- No	63.5% (101/159)	1.00 (reference)	-	1.00 (reference)	-
- Yes	50.0% (42/84)	0.79 (0.62–1.00)	0.05	1.00 (0.82–1.24)	0.94
Ever had oral sex?					
- No	59.9% (109/182)	1.00 (reference)	-		
- Yes	54.8% (34/62)	0.92 (0.71–1.18)	0.50		
Ever had sex while drunk?					
- No	48.5% (32/66)	1.00 (reference)	-	1.00 (reference)	-
- Yes	62.6% (112/179)	0.77 (0.59–1.02)	0.07	0.89 (0.72–1.10)	0.29
Condom as contraceptive?					
- No	30.9% (30/97)	1.00 (reference)	-	1.00 (reference)	-
- Yes	76.9% (113/147)	2.49 (1.82–3.39)	**<0.01**	2.02 (1.53–2.66)	**<0.01**
Hormonal contraceptives?					
- No	60.0% (102/170)	1.00 (reference)	-		
- Yes	55.4% (41/74)	0.92 (0.73–1.17)	0.51		

Education level was significantly associated with condom use. In multivariate analysis, women with Grade 8 to 10 education were approximately 40.0% more likely to report using condoms compared to women with primary school education (p = 0.02); and those with Grade 11 to 12 education were about 50.0% more likely to report using condoms (p<0.01). Women who reported having no partner (single, divorced, widowed or separated) were 1.39 times more likely (95% CI 1.01–1.90) to have used a condom at their last sex act compared to women who were in a stable relationship or married (p = 0.04).

In univariate analysis, age was significantly associated with condom use, with older women being less likely to use condoms. It was no longer significant in the multivariate model, however, most likely because schooling was closely associated with age, with older women being less educated. Age was also associated with perceived choice or insistence on condom use; younger women reported being able to insist on condom use more often than older women. For every five year decrease in age, women were 1.03 times more likely (95% CI 1.01–1.05) to insist on condom use (p = 0.01), adjusting for education level.

Women who reported using condoms as a method of contraception were twice as likely to have used a condom at their last sex act compared to women who did not use condoms as contraception (RR 2.02, 95%CI 1.53–2.66, p<0.01). However, women who said they were using hormonal methods as a contraception method were no more likely to have used a condom at their last sex act compared to women who were not on hormonal contraception (p = 0.51).

## Discussion

This study provides information about the behavioral characteristics, level of HIV knowledge and predictors of condom use in a population of high-risk women in KwaZulu-Natal, South Africa. Specifically, this study illustrates that knowledge of HIV and HIV transmission does not correlate with high condom use in this population. These behavioral risk data are also supported by high STI and HIV infection rates in this cohort over time [29]. Previous studies also illustrated that lack of HIV specific knowledge does not explain low rates of condom use [7], [8], [11], [12]. Of note, there was lower awareness of STI and HIV transmission risk with oral and anal sex, suggesting that this would be an important opportunity for more targeted education.

In our cohort, an important finding is the higher reported efficacy of condom use choice at last sexual encounter with casual rather than steady partners. Similar observations were made in other studies from South Africa and the proposed explanations include concern about perceived lack of trust and threat to intimacy and commitment between steady partners [12], [13], [14], [15], [30]. This highlights the fact that negotiating condom use with steady partners is challenging as it may represent infidelity or, conversely, that non-use of condoms may signal trust. Ironically this means that steady sexual partnerships may represent greater risk of HIV infection, for either partner.

In our cohort, several factors were significantly associated with reported condom use during last sexual encounter. We found an association between education level and increased frequency of reported condom use, a result consistent with other studies that show an association between schooling and reduced levels of HIV risk, particularly in young women [31]. Our results also suggests that using condom for contraceptive purpose seems to be a more acceptable motivation, and women are more likely to use condoms in their sex acts if this is being done as the primary means of contraception. It remains to be seen whether or not use of hormonal contraception in this social context would undermine condom use.

Importantly, we noted that women who perceived that they are able to make a choice about using a condom were twice as likely to report using one. It is therefore important to focus on female empowerment in sexual relationships. Previous literature suggests that encouraging self-agency and endorsing egalitarian gender roles in sexual relationships may have a positive impact on rates of condom use [22]. Similarly, a recent preliminary analysis from a randomized trial in South Africa has shown that interventions to empower a woman's ability to negotiate condom use can lead to an increase in self-reported condom use at the last sex act [32], [33].

Negotiating condom use for women in our cohort is further complicated by the social and cultural context in which these relationships are developed. For although, it has been shown that women have positive attitudes toward condom use [11], South African men are traditionally the decision makers, especially regarding issues of sexual practice [12], [15], [24], [26], [27], [34], [35]. Therefore, despite high levels of knowledge about HIV transmission and prevention, some women are not able to protect themselves from infection.

A strength of this study is the prospective design, with a relatively large cohort, and the high response rate with almost all participants responding to each question. These findings are thus important as they represent unique data for a large cohort of HIV negative women at high risk for HIV infection, who had a broad range of risk factors, including both sex work and non-sex work partners. We believe these data contribute to greater understanding of the sexual networks and risk behaviors in this specific context. Furthermore, this level of data is particularly important in designing culturally relevant risk reduction counseling messages in the context of, for example, HIV prevention studies.

A limitation of the study is that the HIV knowledge and behavioral risk questionnaires were only administered at baseline in the HIV negative cohort, limiting the analysis to cross-sectional data. For example, since data about factors associated with condom use are collected at a single time point, it is not possible to determine causality; prospective study would be needed to determine whether factors such as greater access to formal education would empower the woman to negotiate for condom use. Another notable weakness, shared by much behavioral research, is that the data are self-reported and therefore subject to reporting and recall bias. Attempts were made to minimize reporting bias by making the questions neutral and assuring participants of confidentiality.

### Conclusions

Women at high-risk of HIV infection were found to have had a high level of knowledge about HIV acquisition and prevention of transmission. This indicates that education campaigns have reached women in need of this information. However, importantly, knowledge about the high risk of transmission during anal sex was lacking. Future HIV/AIDS educational campaigns therefore need to include messages regarding the risk of transmission via anal sex.

The self-perceived ability to choose to use condoms is significantly associated with reported condom use at last encounter, and furthers the argument that social barriers, particularly gender power imbalances in relation to sexual decision-making, as opposed to lack of knowledge, may be influential factors preventing safer sexual practices in South Africa. This information highlights the importance of female empowerment in prevention efforts. In particular, and supporting this empowerment hypothesis, our data show that women who attained higher levels of formal education, and those who choose to use condoms for contraception, are more likely to report that they use condoms during sex. Our findings, from a unique cohort of women at high risk for HIV infection with a range of partner types, suggest that focusing on ensuring higher levels of formal education in women and de-stigmatizing condom use, for example by emphasizing condom use as contraception, particularly among men who may view condom use for STI prevention as an indication of infidelity or a lack of trust, are essential components of HIV prevention efforts and promoting safer sexual practices in this part of the world.
